# A Comprehensive Review of the Equine Gut Microbiome in Health and Disease

**DOI:** 10.3390/vetsci13070659

**Published:** 2026-07-07

**Authors:** Aaron C. Ericsson

**Affiliations:** 1 Pathobiology and Integrative Biomedical Sciences, College of Veterinary Medicine, University of Missouri, Columbia, MO 65201, USA; ericssona@missouri.edu or mumetagenomics@missouri.edu; 2 University of Missouri Metagenomics Center, University of Missouri, Columbia, MO 65201, USA

**Keywords:** horse, equine, microbiome, microbiota

## Abstract

Molecular microbiology has revolutionized our understanding of the complex host-associated microbiomes required for normative development and physiology. Horses and other members of the family *Equidae* are particularly reliant on the early maturation and lifelong maintenance of an unusually rich hindgut microbiome for optimal digestion and overall health and performance. Research on the equine gut microbiome has accelerated in the past several years, necessitating a renewed appraisal of the field. The present work is a comprehensive and critical review of the literature regarding the bacterial gastrointestinal microbiome of horses. First, the developmental trajectory of the foal gut microbiome is discussed, followed by descriptions of the taxonomic membership of the core equine gut microbiome, its primary functions and effects on host physiology, and intrinsic and extrinsic factors that shape the equine microbiome during health, with a focus on diet and supplements. Next, evidence supporting adverse effects on the equine gut microbiome of gastrointestinal conditions including colic and colitis, extraintestinal conditions including obesity and laminitis, and pharmacological interventions including antibiotics and non-steroidal anti-inflammatory drugs is summarized. Lastly, clinical and experimental research investigating the effects of treatments targeting the gut microbiome of horses, including probiotics, prebiotics, and fecal microbiome transfer, is critically examined. Conclusions summarize the connection between natural (i.e., wild) equine behavior and the health of the equine gut microbiome and the impacts of human management.

## Simple Summary

The bacteria present in the gut of healthy horses is critical to many aspects of their health. There has been a recent increase in research focused on the gut bacteria of horses to better understand how gut bacteria are affected by diet, supplements, medications, disease, and other factors. Additional research has investigated ways to change the gut bacteria of horses in order to improve their health. The present review is intended to integrate the new and prior literature to produce a concise summary of our current knowledge of gut bacteria in horses and serve as a comprehensive resource for readers of the primary literature on the topic.

## 1. Introduction

The following is intended to serve as an up-to-date and comprehensive review of our understanding of the gastrointestinal microbiome of horses and other *Equidae*, viewed through the lens of next-generation sequencing and molecular microbiology. While the field is sufficiently “long in the tooth” to have produced several excellent review papers [1,2,3], the current effort was inspired by a recent surge in publications and novel findings not captured in previous reviews, as roughly one third of the references were published in 2023 or later. The current review will focus primarily, but not exclusively, on bacterial communities in the gastrointestinal tract (GIT). The equine microbiome also contains fungal, viral, protozoal, or other (e.g., helminth) classes of organisms; however, the bacterial component of the equine microbiome dwarfs the other classes in terms of biomass and has an arguably greater influence on host physiology. Interested readers are referred to studies performing multi-kingdom characterizations of the equine gut microbiome for more information [4,5,6,7,8]. Moreover, the equine microbiome in toto includes microbial communities present in the GIT as well as other anatomical sites including the skin, oral cavity, respiratory tract, ocular surface, and reproductive organs, among others. Again, the biomass contained in the GIT outnumbers all other microbiomes combined by several orders of magnitude and likely has a much greater impact on overall host health than other microbiomes, notwithstanding potential effects of other microbiomes at their site of colonization (e.g., lung microbiome in equine asthma [9], ocular microbiome in ulcerative keratitis [10]). As above, readers are referred to work exploring the microbiome present on the ocular surface [11], skin [12,13,14], reproductive tract [15,16], oral cavity and upper airways [9,17,18], peripheral circulation [19,20], and lungs [21,22,23] of horses for additional information. Lastly, the current review focuses on data generated directly from host-derived samples, as opposed to data generated in silico or using artificial digestive systems, to limit further expansion of an already sizeable work. Efforts are made to use accepted Latin taxonomies, including the recently revised phylum-level assignments [24], when describing previous findings. For example, in studies wherein *Firmicutes* or *Proteobacteria* were originally reported, they are referenced herein as *Bacillota* and *Pseudomonadota*, respectively.

## 2. Methods

### 2.1. Literature Search

A thorough and extensive search of the relevant scientific literature was performed manually using Google Scholar on multiple dates between December of 2025 and June of 2026. Search terms included equine, horse, microbiome, microbiota, 16S rRNA, metabarcoding, and metagenomic, in varying combinations, from any time period. All studies using sequencing-based metabarcoding or metagenomic methodologies were included, with the exception of publications that were not publicly available or were focused purely on technical aspects of sample collection or analysis.

### 2.2. Image Generation

**Figure 1:**
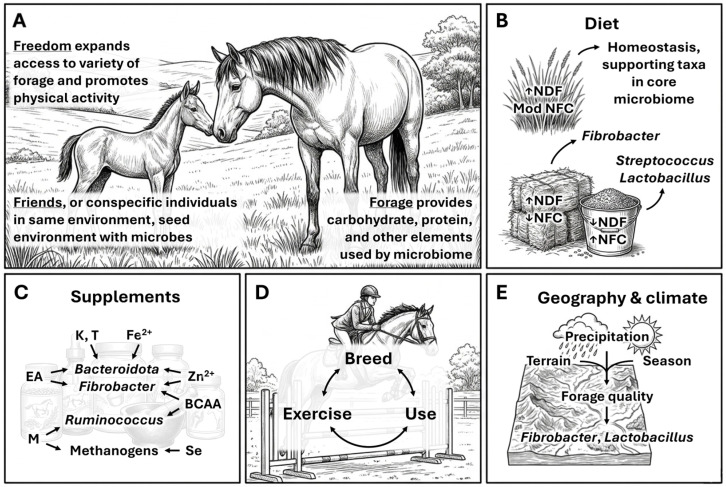
Images depicting key factors underlying gut health in horses, and management-related factors known to influence the composition of the equine gut microbiome. (**A**) Gut microbiological health in horses is predicated on normal acquisition and maturation during the first months of life, and a lifestyle typified by forage, freedom, and friends (the 3 Fs). Forage provides the essential substrates for microbial metabolism, freedom allows access to novel forage and reduces psychological stress, and friends help maintain the microbiological load in the environment and further reduce psychological stress. Factors shaping the composition of the adult microbiome include the amounts of neutral detergent fiber (NDF) and non-fibrous carbohydrates (NFC) in dietary grasses and forage, hay, and cereal grains (**B**); nutritional and health supplements including lysine (K), threonine (T), methionine (M), branched-chain amino acids (BCAA), ellagic acid (EA), selenium (Se), iron (Fe^2+^), and zinc (Zn^2+^) (**C**); interconnected factors like exercise, use, and breed (**D**); and geography, climate, and other environmental variables that ultimately shape the nature and quality of forage and the microbes dependent on them (**E**).

**Figure 2:**
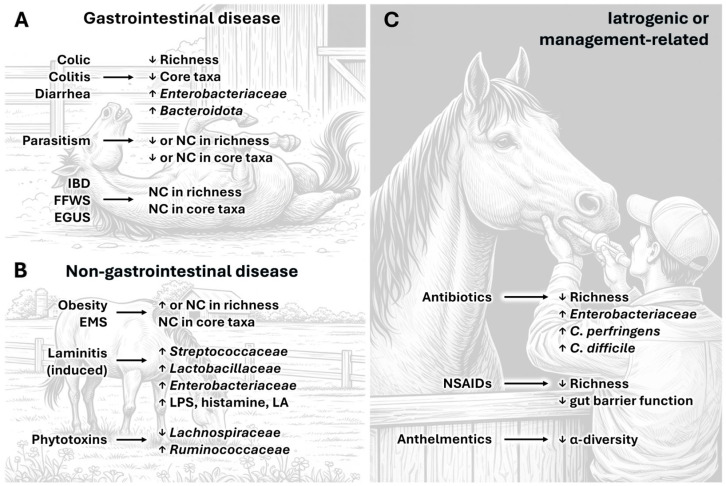
Summaries of characteristics of the most common causes of dysbiosis including (**A**) severe dysbiosis in gastrointestinal conditions including colic, colitis, and diarrhea with less severe or minimal change in other conditions; (**B**) non-gastrointestinal conditions including obesity, laminitis, and other conditions; and (**C**) iatrogenic and management-related practices including administration of antibiotics, non-steroidal anti-inflammatory drugs (NSAIDs), and anthelmentics. NC = no change, LA = lactic acid.

**Figure 3:**
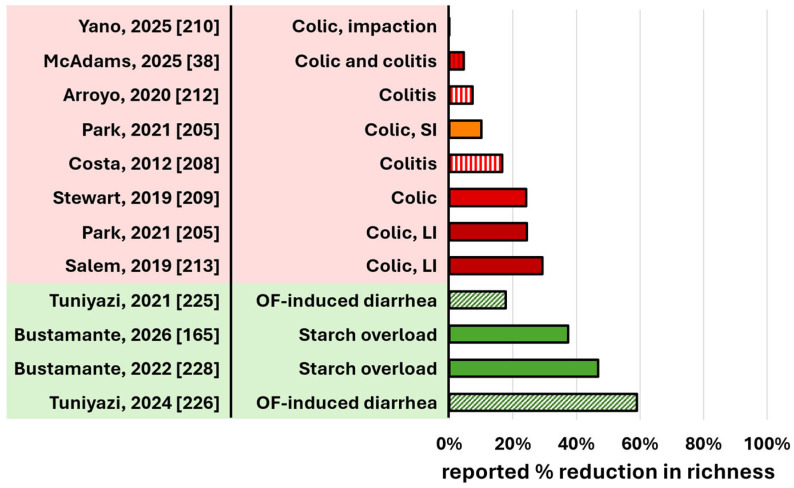
Chart showing the reported percent reduction in richness of the microbiome in horses diagnosed with various forms of colic, including colic restricted to the small intestine (SI) or large intestine (LI), or colitis (pink background), and horses developing diarrhea due to oral administration of oligofructose (OF) or starch overload (green background).

**Figure 4:**
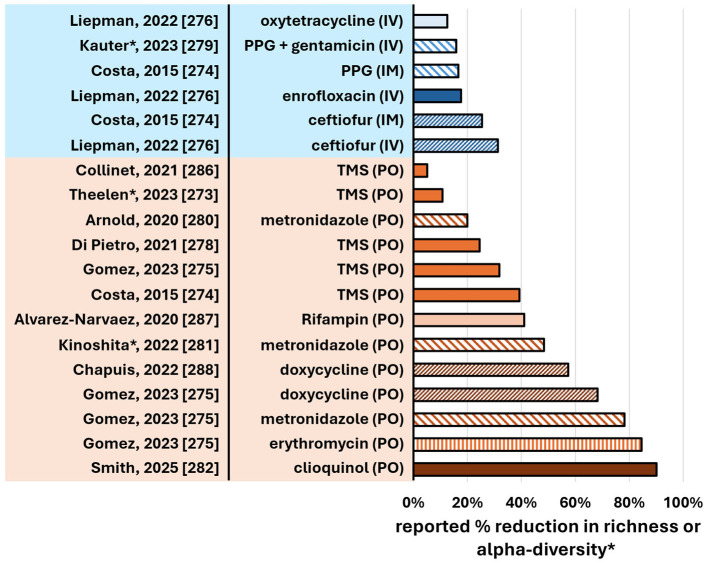
Chart showing the reported percent reduction in richness or alpha-diversity of the microbiome following administration of the listed antimicrobials in studies investigating the influence of antimicrobials on the fecal microbiome of horses. Cool colors represent parenteral drugs, and warm colors represent oral drugs. * denotes that richness was not reported, so comparisons were made using Shannon’s index.

Figure 1 and Figure 2 were generated using FigureLabs (chat.figurelabs.ai) accessed on 22 June 2026. Figure 3 and Figure 4 were made using Microsoft Excel 365 and data reported in the listed references. When specific values were not reported, approximations were made based on published figures.

## 3. Seeding and Maturation of the Equine Microbiome

While data suggest equine meconium may harbor low-biomass microbial populations originating from the amniotic fluid [25], true colonization of the foal hindgut begins rapidly in the immediate neonatal period, with a several log-fold expansion of bacterial cell numbers in the first 24 h of life [26]. The foal hindgut is seeded by bacteria from the mare’s milk, vaginal canal, oral cavity, and environment [26,27,28]. Facultative anaerobes such as lactic acid bacteria (LAB, e.g., *Lactobacillus* and *Streptococcus*) and *Enterobacteriaceae* are present at high relative abundance in the neonatal foal hindgut [29,30]. The foal gut microbiome matures into an apparently adult composition between two and three months of age [27,29,31,32,33,34,35], in large part due to coprophagy by the foal [36]. This involves expansion and diversification of a rich hindgut core microbiome of obligate anaerobes comprising members of the phyla *Bacillota*, *Bacteroidota*, *Kiritimatiellaeota* (i.e., *Verrucomicrobia* subdivision 5) [37], *Fibrobacterota*, *Actinomycetota*, *Spirochaetota*, *Pseudomonadota*, and *Methanobacteriota* [6,38]. Adjusted for mean lifespan, this process occurs much earlier in horses than in other hosts, long before the typical age of weaning. In Table 1, Lifespan-adjusted Time to Maturation (LTM) represents the percentage of the average lifespan that has elapsed at the time of hindgut microbiome maturation in different mammalian species.

Relative to progressive weaning with increasingly longer durations away from the mare over the course of a month, abrupt weaning at 8 months of age results in greater stress in the foal (based on salivary cortisol 24 h post-weaning) accompanied by a transient increase in RA of *Streptococcus* that normalizes by one week post-weaning [51]. The final taxonomic changes that occur during weaning are accompanied by maturation of the metabolic capacity of the microbes [52].

### 3.1. Upper Gastrointestinal Tract Microbiome

Following maturation of the hindgut, regions within the upper gastrointestinal tract (GIT), including stomach, duodenum, jejunum, and ileum, develop their own distinct compositions, often retaining characteristics of the early-life microbiome. Thus, in adulthood, the composition of the equine gut microbiome varies along the course of the gastrointestinal tract (GIT) [4,53,54,55,56,57,58,59,60]. The difference between the bacterial microbiome in the upper and lower GIT of horses is dramatic. Compositional differences between regions within either the upper or lower GIT are more subtle, but significant, nonetheless [61]. The microbiome present in the upper GIT of horses is dominated by LAB (e.g., *Lactobacillus*, *Enterococcus*, *Streptococcus*) and a handful of other *Bacillota* (*Veillonella*, *Sarcina*), *Pseudomonadota* (e.g., *Moraxella*, *Actinobacillus*, *Acinetobacter*), and *Bacteroidota* (*Porphyromonas*, *Alloprevotella*) [54,62,63] and is more variable between horses than the hindgut [53,54,64].

### 3.2. Lower Gastrointestinal Tract Microbiome

Presumably, the slower transit and large volume of the lower GIT (cecum and colon) allow time for communities to equilibrate and normalize. While the cecum has the greatest bacterial density within the hindgut, the right colon has the greatest bacterial richness and diversity, the highest protozoal and fungal biomass, and the highest concentrations of total volatile fatty acids (VFAs) [4,65]. There is a desire to precisely quantify and catalog the members of the healthy hindgut within the equine population to serve as a reference interval against which other samples can be compared, better understand cross-feeding networks, and inform clinical and molecular studies targeting specific taxa.

Thus, several groups have characterized the core fecal microbiome [66] within various cohorts, defined as taxa reaching specific thresholds of prevalence and mean relative abundance (RA). Despite differences in horse populations and a host of technical details including prevalence and RA thresholds, outcomes of these studies align surprisingly well. Comparing the results of several studies employing multiple different sequencing platforms and 16S rRNA regions [6,7,30,38,67,68,69,70,71,72,73,74], collectively comprising data from over 2350 healthy horses in six different countries, over 100 genus/group-level taxonomies were identified as part of the core equine microbiome in more than one study (Table S1).

Genera or uncultured groups found in more than one of the included studies [6,7,30,38,67,68,69,70,71,72,73,74] come from roughly a dozen phyla, with the greatest phylogenetic diversity coming from the phyla *Bacillota* and *Bacteroidota*. The most diverse families within *Bacillota* in the fecal microbiome include *Lachnospiraceae*, *Ruminococcaceae*, *Oscillospiraceae*, *Erysipelotrichaceae*, and *Clostridiaceae*. Families *Streptococcaceae* and *Lactobacillaceae* are also considered part of the core fecal microbiome in healthy horses. Within the *Bacteroidota*, dominant families in the horse fecal microbiome include *Prevotellaceae* and several uncultured taxa within the *Bacteroidales* including groups BF311, BS11, F082, p_251-o5, and RF16. Other phyla within the core microbiome of the equine hindgut are less phylogenetically diverse, often represented by one or two genera. These include *Fibrobacterota* (*Fibrobacter*), *Spirochaetota* (*Treponema*), *Kiritimatiellaeota* (WCHB1-41 and RFP12 groups), *Verrucomicrobiota* (*Akkermansia*), *Mycoplasmatota* (*Anaeroplasma*, *Mycoplasma*), and *Actinomycetota* (families *Coriobacteriaceae* and *Eggerthellaceae*). The core microbiome is reported at the taxonomic level of family or genus in order to assemble a manageable catalog of microbes. In truth, several different species or strains of bacteria within a given genus are often present in equine feces. With the benefit of full-length 16S rRNA sequencing, multiple species of *Blautia*, *Roseburia*, *Ruminococcus*, and several other *Bacillota* are detected at high prevalence in the fecal microbiome of wild and Thoroughbred horses [7].

## 4. Functionality of the Equine Gut Microbiome

Mammalian host-associated microbiomes consist of multiple overlapping syntrophic relationships, resulting in complex networks of cross-feeding [75]. Many microbial processes in the hindgut occur through the sequential activity of numerous unrelated genera. For example, carbohydrate fermentation is accomplished by taxa from numerous families, each possessing a unique repertoire of carbohydrate-active enzymes (CAZymes) needed collectively to fully catabolize complex polysaccharide molecules into energy sources for the host such as butyrate and acetate [76]. Multiple parallel metabolic pathways often converge on the same end metabolite, as is the case with microbial butyrate synthesis, achieved through four separate fermentative pathways [77,78,79]. As such, microbial richness (i.e., the total number of different species) is generally associated with gut health and overall host fitness. Microbes capable of converting metabolic end products of the host or other microbes fill a niche and enhance overall process efficiency. For example, lactic acid produced by skeletal muscle during exercise (and by LAB) can be used by *Veillonella* to generate propionate and fix inorganic carbon into its organic form. *Veillonella* spp. are enriched in the feces of endurance runners post-marathon [80]. In cattle, probiotics containing *Megasphaera elsdenii*, another member of the *Negativicutes* related to *Veillonella*, are associated with improved fermentation efficiency characterized by increased SCFA production and reduced lactic acid and methane, enhanced average daily gain and body condition score (BCS), and improved overall health [81]. Likewise, methanogenic Archaea, which consume H^+^ ions to generate ATP, enhance the overall fermentative capacity of the microbiome. In a six-year study with 794 feral horses, the methanogenic capacity of the microbiome was associated with increased survival [82].

The microbiome in each region of the gut provides diet- and region-dependent functions and signals to the host. For example, short-chain fatty acids (SCFAs) and other FAs resulting from bacterial fermentation of fiber stimulate GPR41/43 expressed on L cells in the distal ileum and colon. Additionally, while certain microbial functions (e.g., fermentation of carbohydrates) or host–microbe interactions (e.g., stimulation of mucosa-associated lymphoid tissue, MALT) may occur predominantly in one specific region of the GIT, they are not necessarily restricted to that region. For example, fermentation of fructans and simple sugars begins rapidly in the stomach [83] and upper GIT [84], while more complex polysaccharides are fermented in the hindgut [85]. Similarly, induction of different immune cell populations by the gut microbiota likely occurs differently in the upper and lower GIT [57,86]. Thus, similar to host physiology, the microbiome in each region is somewhat specialized to function in that environment, but there is also overlap between regions.

### 4.1. General Functions of the Microbiome

A comprehensive and detailed description of the functions performed by the equine gut microbiome is beyond the scope of the current review. However, several key microbial processes or functions that are essential to host health merit discussion. Host benefits from these processes can occur locally (e.g., the regulation of cell turnover and inflammation by butyrate in the GIT), or in distant locations via cell-mediated and humoral mechanisms.

#### 4.1.1. Fermentation

Based on metagenomic gene content within the equine hindgut, carbohydrate metabolism is the principal metabolic pathway in the cecum, followed by amino acid and then nucleotide metabolism [87,88,89]. The CAZymes associated with carbohydrate fermentation (arranged in clusters known as polysaccharide utilization loci, PULs) map primarily to *Bacillota* and *Bacteroidota*, albeit with substantial presence also detected in *Verrucomicrobiota*, *Spirochaetota*, *Pseudomonadota*, and *Fibrobacterota*. Glycosyl hydrolases (GH) and glycosyl transferases (GT) represent the dominant classes of CAZymes in the equine hindgut, followed by carbohydrate-binding molecules and carboxyesterases [87,88,89,90,91]. GH catabolize carbohydrates via hydrolysis of glycosidic linkages, and many are dependent on Zn^2+^, Cu^2+^, or other metals [90,92,93,94]. VFAs represent the dominant and most-studied end-products of carbohydrate fermentation, although a wide array of other bioactive compounds are also produced. Outstanding and detailed reviews of prokaryotic fermentation in general [95] and in the equine hindgut [76] are recommended for curious readers.

#### 4.1.2. Vitamin Synthesis

While dietary sources provide the majority of B vitamins and vitamin K1 absorbed by the host, the gut microbiome also synthesizes a substantial portion, often in a sequential and cooperative fashion similar to fermentation [96]. Several microbial enzymes involved in vitamin synthesis in the gut also require trace metals as co-factors [97]. While limited research has been performed investigating vitamin synthesis in the equine hindgut, an excellent review of vitamin synthesis in the human gut provides additional relevant information [98].

#### 4.1.3. Colonization Resistance

Healthy gut microbiomes also prevent the colonization or expansion of newly introduced microbes, including pathogens. This occurs via direct mechanisms, wherein commensal microbes produce bacteriocins and other compounds to inhibit the growth of other microbes, and indirect mechanisms, wherein commensal microbes induce host immune responses that inhibit growth or colonization of other microbes [99]. The process begins in the neonate via acquisition of protective bacteria from the mare in the colostrum and milk [27,28] and is augmented during early life by coprophagic behavior [36]. Notably, segmented filamentous bacteria (SFB, *Candidatus Savagella*) have been reported in the adult equine ileum [100]. Host-specific SFB are found in all healthy vertebrates under normal conditions [101], and their presence is associated with significantly increased production of IgA and induction of CD4^+^ T_H_17 immune responses [102,103], increased epithelial production of antimicrobial peptides, and enhanced colonization resistance against pathogenic *Enterobacteriaceae* in multiple host species [102,104,105]. Several findings suggest the microbiomes in the upper and lower GIT are both involved in immune homeostasis in horses. First, luminal levels of IgA are higher in the upper GIT than the cecum or colon of horses, despite greater numbers of IgA^+^ cells in the cecum [57]. Second, the RA of various members of the core microbiome in both the ileum and cecum correlate significantly with the expression of several anti- and proinflammatory cytokines at each site [86]. Lastly, while attempts to predict susceptibility to *Rhodococcus equi* pneumonia based on the richness of the microbiome at roughly one month of age were unsuccessful [106], the richness of the foal microbiome of horses at 1 to 2 months of age is negatively associated with mortality from infectious respiratory disease among other conditions [33].

#### 4.1.4. Bile Acid Metabolism

Since horses do not have a gallbladder, conjugated bile acids (BAs) are released directly into the lumen of the duodenum. Conjugated BAs have potent antimicrobial activity, and bile salt hydrolase (*bsh*) expression is an important defense mechanism against BAs shared by many microbes in the human gut [107]. Enzymatic activity of *bsh* results in deconjugation of BAs, decreased solubility, increased passage to the hindgut, and susceptibility to 7α-dehydroxylation by genes within the bile acid-induced (BAI) operon of taxa from families *Lachnospiraceae* and *Peptostreptococcaceae* [108]. In addition to their activity as detergents in the gut, primary and secondary BAs signal through G protein-coupled bile acid receptor 1 (*Gpbar1*, i.e., TGR5) in the GIT, liver, and adipose tissue to regulate energy expenditure and insulin sensitivity. Notably, changes in plasma levels of certain BAs are significantly associated with insulin dysregulation in horses [109]. Receptors in the gut communicate with the central nervous system via vagal afferents [110]. These signals contribute to the effects of diet on the gut microbiome, production, and behavior [111]. 

#### 4.1.5. Xenobiotic Transformation

The term xenobiotic generally refers to chemical substances present in the body that are foreign and not naturally produced, including drugs, food additives, and environmental pollutants. Gut microbial communities influence the host exposure to xenobiotics through a wide range of transformation reactions, conserved among vertebrate microbiomes [112,113]. As such, the equine microbiome may be responsible for the variability in clinical response to many drugs.

#### 4.1.6. Associations with Performance

Given the characteristics of the microbiome of elite human athletes [80], there is interest in the relationship between the microbiome and performance in horses. To be clear, this is a dynamic and complex relationship between exercise, BCS, the microbiome, and performance. Regarding the acute effects of exercise on the equine gut microbiome, they are likely modest. While several studies have failed to detect differences between samples collected pre- and post-exercise [114,115,116], others report changes in the microbiome including increased alpha-diversity [117] and increased RA of several genera within the *Bacteroidota* phylum [118,119]. That being said, diet and exercise sufficient to result in weight loss are associated with subtle changes in the microbiome [120,121]. Conversely, chronic food intake sufficient to induce weight gain (e.g., feeding 180% of maintenance metabolizable energy requirements for two years) is associated with a decrease in richness and reduced RA of *Fibrobacter* [122].

High- and low-performing horses in response to a nine-month conditioning regimen differ in the RA of multiple genera within the phylum *Bacteroidota* (*Bacteroides*, *Parabacteroides*, and *Prevotella*) and certain CAZymes involved in fermentation [123]. Similar taxa are enriched in the microbiome of horses following a 30-day riding training program, when compared to an untrained control group [118]. Further supporting the relationship between microbial richness, energy harvest, and equine performance, the metagenomic content of elite endurance athlete horses is a strong predictor of cardiovascular fitness [124]. While not all studies have been able to identify microbial biomarkers of equine performance [125], several have identified microbial taxa and metabolites (e.g., acetate and butyrate) that are positively associated with performance [89,124,126,127,128]. Reflecting the relationship between richness and function, the metabolic pathways enriched in racehorses comprise contributions from a large and phylogenetically diverse collection of microbes rather than a limited number of related taxa [89]. Notably, the richness of the early life microbiome of horses, before and during the point of maturation (i.e., 1 to 2 months of age), is positively associated with multiple metrics of athletic performance in the first three years of life [33].

## 5. Factors Influencing the Healthy Adult Equine Microbiome

### 5.1. Intrinsic Factors

As in other host species, the gut microbiome of a healthy horse is shaped by intrinsic factors including genome (e.g., breed, sex), age, and a host of extrinsic environmental factors including (but not limited to) diet, geography, occupation, and psychological stress (Figure 1). As described above, the hindgut of a foal is fully developed long before becoming a yearling. Beyond that point and throughout adulthood, left unperturbed, healthy horses maintain a consistently rich hindgut microbiome comprising over 1000 distinct taxa [129]. As in humans [130] and rodents [131], the microbiome of horses undergoes characteristic changes during aging. Richness and alpha-diversity (i.e., the evenness of the bacterial community) are reduced in elderly horses. Several studies indicate this decline begins around 20 years of age [132,133,134,135]. LPS-expressing *Pseudomonadota* are also reportedly enriched in the microbiome of elderly (i.e., >19 years) horses [69,132], of potential relevance to the observed changes in immune activity in aged horses [136]. The effects of sex or sex hormones on the fecal microbiome of adult horses are significant but modest relative to other factors and inter-individual variability [38,137,138,139,140]. Alternatively, the species and breed of equids have a strong influence on the fecal microbiome composition [38,70,135,141,142,143,144,145].

### 5.2. Dietary and Related Factors

Regarding extrinsic factors, diet is arguably the most studied factor, often as an unavoidable covariate. For example, captivity is associated with reduced taxonomic and metabolic diversity in the equine gut [30,146,147,148], presumably by restricting access to varied forage. Placed on different diets, the fecal microbiome of horses will respond rapidly to the available substrates and undergo diet-associated compositional changes [149,150,151]. While the majority of research focuses on responses in the hindgut due to the convenience of fecal sampling, the upper and lower GIT microbiomes are both responsive to dietary content [64,152]. Following transition from pasture to concentrate or vice versa, the composition of the fecal microbiome shows evidence of change in as little as 24 h, but the full response to a new diet occurs over several days [153,154]. Independent of macromolecular content, other aspects of feeding management can influence the equine fecal microbiome. For example, provision of dietary content in the form of total mixed rations (TMR) is associated with increased daily weight gain and nutrient digestibility and significantly increased RA of *Treponema* [155,156]. Similarly, provision of an equivalent amount of concentrate as a single meal results in significantly greater cecal RA of *Streptococcus*, *Coprococcus*, and *Prevotella* relative to horses consuming the same amount of concentrate divided into two or three meals [157].

#### 5.2.1. Carbohydrates

Hay contains high levels of neutral-detergent fiber (NDF, cellulose, hemicellulose, and lignin). Consumption of hay and haylage is associated with an increased RA of *Fibrobacter* in multiple studies [64,134,158,159]. Grass hays typically contain higher NDF relative to legume hays, and *Fibrobacteraceae* is accordingly enriched in the cecum and feces of horses fed brome grass relative to alfalfa [160]. In contrast, cereal grains such as corn, barley, wheat bran, and oats are much higher in non-fibrous carbohydrates (NFC, starch, simple sugars, fructans). Addition of NFC to an alfalfa hay diet is associated with a dose-dependent decrease in RA of *Fibrobacteraceae* and increase in RA of *Streptococcus* [161]. Supplementation of hay-based diets with other sources of NFC including barley or high-starch concentrates is similarly associated with decreased RA of *Fibrobacter* [162] and increased RA of *Streptococcus* [64,163,164,165,166] in the equine hindgut. Other consistent changes in the fecal microbiome associated with increased dietary NFC include enrichment of members of family *Succinivibrionaceae* [64,134,166,167,168,169,170] (phylum *Pseudomonadota*) and several closely related genera from phylum *Bacteroidota* (e.g., *Prevotella*, *Alloprevotella*, *Paraprevotella*) [129,167,168,171,172], as well as decreased RA of several members of phylum *Bacillota* [168,171,172]. It is worth noting that different sources of NFC such as oats, corn, and barley may have distinct effects on the equine microbiome [173]. Supplementation with sources of NFC is also associated with increased VFA production [160,161,167,169,172], decreased hindgut pH [161,167,169,172], and a greater growth rate [168,171].

#### 5.2.2. Protein

Comparisons of different amounts of dietary crude protein (CP) suggest that CP of roughly 12% is associated with optimal alpha-diversity and composition of the fecal microbiome, as horses receiving lower (10.85%) or higher (13.25%) CP were associated with decreased alpha-diversity and increased RA of *Acinetobacter*, while horses receiving 12% were associated with higher RA of several members of the core microbiome within *Bacillota* and *Bacteroidota* and optimal nutrient digestibility [174]. A second study comparing roughly 11%, 12.5%, and 14% CP in Dezhou donkeys reported similar findings with optimal protein digestibility at 12.5% and significant associations between CP content and the RA of several *Bacillota* including members of *Oscillospiraceae* UCG-002 and UCG-005 and *Ruminococcaceae* NK4A214 group [175].

#### 5.2.3. Effects of Environment on Forage

Meteorological and geographic factors affecting the carbohydrate content of forage would thus be expected to influence the hindgut microbiome of herbivores. Accordingly, seasonality and the subsequent availability of seasonal forages and silage are associated with changes in the equine gut microbiome [7,150,159,176,177,178,179], and regional and farm-level geography have a substantial effect on the fecal microbiome of adults and foals [31,38,142,146,180,181,182]. For example, warm-season grasses, which are typically higher in fiber than cool-season grasses, are associated with greater RA of *Fibrobacterota* and lower RA of *Lactobacillaceae* [183]. Ultimately, the specific property on which a horse or donkey resides represents a dominant variable in shaping its microbiome [182]. That being said, the healthy equine microbiome is often resilient to diet changes, and responses are individualized [184,185].

### 5.3. Dietary Supplements

Over two-thirds of horse owners or caretakers provide supplements to their horses, based on a general sense of safety or the recommendation of a veterinarian [186,187]. While there is still a lack of high-quality data regarding efficacy of many supplements [188], there is a rapidly growing body of research supporting their clinical benefits and documenting their effects on the microbiome. Dietary supplements can be classified according to their purported health benefit, including those targeting joint health, hoof or coat health, digestive health, nervous system and neuromuscular health, and antioxidant or anti-inflammatory properties. Ingredients in equine supplements include a wide range of oils and fatty acids, amino acids, protein, vitamins, trace minerals, and botanical compounds. Note that probiotic and prebiotic supplements are discussed further below in Section 6.

#### 5.3.1. Omega-3 Polyunsaturated Fatty Acids

The health benefits of omega-3 polyunsaturated fatty acids (PUFAs) including eicosapentaenoic acid (EPA), docosahexaenoic acid (DHA), and alpha-linolenic acid (ALA) are well-established in humans. While the balance of omega-3 to omega-6 PUFAs is of interest in equine nutrition [189], little research has been published reporting the effects of PUFAs on the equine microbiome. Several recent reviews of the literature in other species agree that omega-3 PUFAs function as prebiotics, contributing to the enrichment of several SCFA-producing bacteria and increased production of SCFAs [190,191,192].

#### 5.3.2. Amino Acids

Methionine, lysine, and threonine are commonly supplemented to promote growth in young horses and maintenance of healthy coat and hooves. Methionine supplementation is associated with increased RA of specific taxa (e.g., *Methanocorpusculum*, *Ruminococcus*) in donkeys [193] while lysine and threonine are associated with enrichment of several members of the *Bacteroidota* (*Prevotellaceae*, *Rikenellaceae*, and p_251_o5) [194]. Branched chain amino acids (valine, leucine, and isoleucine) provided to pregnant mares are associated with enrichment of *Ruminococcus*, *Fibrobacter*, and *Treponema* in foals [195].

#### 5.3.3. Antioxidants and Trace Minerals

Ellagic acid, a polyphenol found in many nuts, fruits, and vegetables, is a natural antioxidant marketed in equine supplements to reduce inflammation and support orthopedic and neurologic health. Supplementation of yearling Thoroughbreds with ellagic acid is associated with significant, dose-dependent increases in weight gain, fiber and protein digestibility, RA of *Fibrobacterota* and *Bacteroidota*, and SCFA production [196]. Supplementation of bioavailable selenium in the form of L-selenomethionine is also associated with significant, dose-dependent increases in fiber and protein digestibility, along with increased RA of *Methanobrevibacter* and *Actinomyces* in Yili mares [197]. Supplementation with Zn^2+^ in the form of zinc chloride hydroxide or zinc methionine is associated with a dose-dependent decrease in richness, decreased RA of *Bacteroidota* and *Fibrobacterota*, and reduced SCFA synthesis [198]. Supplementation with Fe^2+^ (ferrous sulfate monohydrate) at 720 ppm for 30 days is also associated with decreased RA of an unclassified member of the *Bacteroidota*, along with a reduced RA of *Streptococcus* and *Anaeroplasma* and increased RA of *Alloprevotella* [199]. Addition of biochar as a feed additive to adsorb toxins and other harmful compounds has no detectable effect on the hindgut microbiome [200].

#### 5.3.4. Joint Supplements

Glucosamine and chondroitin sulfate, common components in supplements marketed for support of joint health, have limited intestinal absorption, suggesting a microbiome-associated mode-of-action (and explanation for variability in clinical responses). While evidence of effects of glucosamine on the equine microbiome is limited, evidence exists suggesting chondroitin sulfate is associated with the enrichment of the genus *Bacteroides* in other hosts [201].

## 6. Dysbiosis—Causes and Consequences

Dysbiosis, defined as significant change in microbiome composition associated with an adverse health condition or challenge to the microbiome, exists on a spectrum of severity and duration from minor and/or transient to dramatic and/or long-lasting. Dysbiosis can be organic, arising from disease-associated changes in the gut environment, or induced, arising from environmental, iatrogenic, domestogenic, or management-related factors [202] (Figure 2). Disease-associated dysbiosis often occurs in response to the primary disease etiology, but it may then exacerbate the condition. In contrast, iatrogenic and management-related dysbiosis may lead to de novo development of adverse effects on the health and well-being of horses.

### 6.1. Gastrointestinal Conditions

#### 6.1.1. Colic and Colitis

Conditions affecting the gastrointestinal tract are often associated with dysbiosis. The dysbiosis associated with colitis may be of equal or greater severity than that observed during antibiotic-induced dysbiosis [142,203]. The nature and degree of change during colic and colitis depend on the affected region of the GI tract [204,205] and presence or absence of inflammation [38,206] among other factors. Moreover, the duration of the condition influences the severity of dysbiosis, as microbial richness declines over time during unresolved colic, driven by loss of *Bacillota* and concomitant increases in RA of *Pseudomonadota* and *Bacteroidota* [38,204]. Characteristic changes in the gut microbiome of mares, including increased RA of *Enterobacteriaceae* and other *Pseudomonadota*, precede the development of colic, suggesting a causative or contributory role for dysbiosis in post-partum colic [207].

Spontaneous colic, colitis, and diarrhea are characterized by significantly reduced richness and alpha-diversity [38,204,205,208,209,210,211,212,213,214,215]. Colic arising from large intestinal (LI) lesions appear to be associated with a more severe decline in richness, relative to colic from small intestinal (SI) lesions. In studies comprising solely [205,213] or predominantly [209] horses with colic from LI lesions, richness was between 71% and 76% that of healthy controls (Figure 3). In studies comprising solely horses with colic or colitis from SI lesions [205] or horses with mixed lesion sites [38,208,210,212], richness was between 83% and 100% that of controls.

Characteristic changes in beta-diversity associated with colic, colitis, and diarrhea include reduced RA of core families and genera within the phyla *Bacillota* (*Ruminococcaceae*, *Lachnospiraceae*, and *Oscillospiraceae*) [38,204,209,212,213,214,216], *Bacteroidota* (*Prevotella*) [209,213], *Kiritimatiellaeota* (WCHB1-41) [205,210,214], *Fibrobacterota* (*Fibrobacter*) [211,212], and *Methanobacteriota* (*Methanobrevibacter*) [205]. In contrast, taxa enriched during colic, colitis, and diarrhea include other members of the phyla *Bacillota* (LAB, including *Streptococcus*, *Lactobacillus*, and *Enterococcus*) [38,205,209,210,211,212], *Bacteroidota* (*Bacteroides*, BF311, BS11, *Prevotellaceae*) [38,204,209,210,216], *Fusobacteriota* (*Fusobacterium*) [208,212], and *Pseudomonadota* (*Enterobacteriaceae*, including *Escherichia* and *Acinetobacter*) [38,205,207,212]. The depletion or loss of commensal taxa has clinical implications based on their critical role in gut health and may contribute directly to the proliferation of pathogenic microbes [217]. The enrichment of certain taxa is of clinical concern due to their ability to lower gut pH (and downstream effects on SCFA synthesis), their expression of LPS and other inflammatory molecules, and the prevalence of multidrug resistance among them [146].

#### 6.1.2. Gastrointestinal Parasitism

Evidence suggests that the composition of the bacterial microbiome regulates the parasite burden in the equine hindgut. In a study comparing the fecal microbiome of abruptly and gradually weaned foals, positive and negative associations were identified between the RA of different commensal bacteria and fecal egg counts (FEC) [51]. Moreover, horses diagnosed with larval cyathostominosis and showing clinical signs develop dysbiosis characterized by a significant reduction (~20%) in fecal microbiome richness, increased RA of *Streptococcus*, and reduced RA of *Ruminococcus*, *Fibrobacter*, and *Treponema* relative to unaffected horses on the same pasture [218]. Independent of clinical signs, high FEC in horses has been associated with greater alpha-diversity [219,220] and subtle changes or differences in beta-diversity of the fecal microbiome [221,222,223]. Diet has an apparently greater effect, however, as a high-starch diet induces a decrease in alpha-diversity and concurrent increase in FECs [169].

It is worth noting that antibiotic- and oligofructose-induced diarrhea are associated with similar changes, i.e., reduced richness (Figure 3), over-representation of LAB [224], and reduced RA of dominant commensal microbes [142,165,225,226,227,228], suggesting the changes observed in horses affected with colic, colitis, and parasitism may represent, at least partially, common manifestations of impaired GI function or diarrhea.

#### 6.1.3. Inflammatory Bowel Disease (IBD)

There are, however, conditions affecting the GIT, some of which are associated with diarrhea, that do not result in those characteristic changes, including equine IBD, free fecal water syndrome (FFWS), and equine gastric ulcer syndrome (EGUS). At present, there is only one large, well-controlled study comparing horses with IBD (*n* = 49) and healthy pasture- and barn-mates (*n* = 27). Surprisingly, no differences were detected in richness or alpha-diversity between horses with IBD and healthy control. While beta-diversity changed significantly, IBD-associated dysbiosis is apparently characterized by reduced RA of commensal *Bacillota* and increased RA of commensal *Bacteroidota*, rather than expansion of LAB, *Enterobacteriaceae*, or *Fusobacteriota* [229].

#### 6.1.4. Free Fecal Water Syndrome

There has been considerable research investigating changes in the fecal microbiome associated with free fecal water syndrome (FFWS) in horses, a recently recognized and vexing condition characterized by passage of formed fecal material followed by a liquid phase [230]. While the exact etiology of FFWS is unknown, several risk factors have been identified including dietary and stress-related conditions [231,232]. Attempts by five independent groups to identify characteristic features of the microbiome in FFWS-affected horses uniformly failed to detect any differences in richness or alpha-diversity between affected horses and unaffected controls [233,234,235,236,237], and only one identified extremely subtle effects on beta-diversity [237]. Differentially abundant taxa were identified in three of the studies [233,236,237], with some agreement between the two most well-powered including increased RA of *Alloprevotella* spp. in FFWS-affected horses [236,237].

#### 6.1.5. Equine Gastric Ulcer Syndrome

Following the characterization of a fairly diverse and stable gastric microbiome in horses [23,62,238], investigations into the influence of the gut microbiome on EGUS have focused on the gastric microbiome rather than the hindgut microbiome. An initial study comparing Thoroughbred racehorses with mild (*n* = 3), moderate (*n* = 3), and severe (*n* = 2) EGUS and unaffected controls (*n* = 2) failed to detect significant differences in the composition of the gastric microbiome based on disease severity [239]. Rather, the composition of the gastric microbiome was significantly associated with management factors including feeding frequency and type of bedding [239]. Subsequent studies with larger sample sizes identified significant differences in beta-diversity between horses with EGUS and unaffected controls [63] and between matched affected and unaffected tissue biopsies from horses with EGUS [240,241], and confirmed that management-related factors have a substantial impact on the gastric microbiome [242]. Notably, equine athletes that switched to a low-starch diet demonstrated a decrease in the ratio of *Bacillota* to *Bacteroidota* in their feces, which correlated with the degree of improvement in gastric lesion scores [243]. While *Helicobacter* spp. are found at low RA in the equine gastric and fecal microbiomes [63,239,244], no associations with EGUS have been identified.

### 6.2. Conditions Affecting Other Organ Systems

As in humans, numerous conditions affecting other organ systems in horses may result in dysbiosis. In a recent survey of 1190 horses admitted to one of eight different teaching hospitals in three countries, including healthy horses (*n* = 196) and horses presenting with acute and chronic GI conditions, as well as primary complaints affecting the musculoskeletal, ocular, integumentary, respiratory, and other systems, horses admitted for respiratory (*n* = 73) and dental conditions (*n* = 21) demonstrated reductions in richness and alpha-diversity and changes in beta-diversity approaching those observed in horses with acute GI conditions (*n* = 382) [38]. While it is unclear whether these differences are the result of inappetence, inflammation, or other unrecognized variables, they highlight the relevance of the gut microbiome to conditions outside the GIT. This section summarizes our current understanding of the equine microbiome and conditions primarily affecting organ systems outside the GIT.

#### 6.2.1. Obesity and Endocrinopathies

The effects of obesity and its associated metabolic consequences on the equine fecal microbiome are complex. For reasons that are unclear, obesity has been associated with a counter-intuitive increase in richness and diversity in two studies [69,245] and no difference in a third [246], with no consistent effects on beta-diversity relative to non-obese controls. Two studies comparing horses affected with Equine Metabolic Syndrome (EMS), a common sequela to obesity, and healthy controls both failed to detect a difference in richness and found only modest, and not particularly concordant, effects on beta-diversity [247,248]. As suggested elsewhere [249], it may be that horses with insulin dysregulation are more susceptible to changes in diet. In a survey of 16 ponies, including 11 with moderate or severe insulin dysregulation (with normal pituitary pars intermedia function), the evenness of the fecal microbiome was significantly associated (R = −0.738, *p* = 0.001) with post-prandial plasma aGLP-1 [250], an incretin that closely follows insulin levels following a meal [251]. A multi-omic longitudinal comparison of EMS-affected and healthy control horses failed to detect significant differences in the fecal microbiome. However, BAs and other plasma metabolites were able to discriminate insulin-dysregulated horses from controls, and levels of the BA taurochenodeoxycholic acid were significantly correlated with the RA of *Pseudomonadota* [109]. A study of horses diagnosed with pituitary pars intermedia dysfunction (PPID), characterized by loss of inhibitory dopaminergic neurons and subsequent excessive secretion of ACTH, found modest differences in beta-diversity between affected and healthy controls characterized by increased RA of *Fibrobacter* and *Pilobolus* and reduced RA of several members of the core microbiome [178].

#### 6.2.2. Orthopedic Conditions

The most common orthopedic condition linked to the gut microbiome in horses is laminitis. Following experimental induction of laminitis via oligofructose, the fecal and cecal microbiomes change rapidly, including enrichment of *Streptococcus* and *Lactobacillus* [225,252,253,254]. Specifically, *Streptococcus* proliferates rapidly prior to the onset of laminitis, driving secondary increases in the RA of *Lactobacillus*, *Negativicutes* like *Veillonella* [254] and *Megasphaera* [225] capable of metabolizing lactic acid, and *Enterobacteriaceae* [252,253]. During oligofructose-induced laminitis, levels of histamine, lactic acid, and LPS are all elevated in horse serum [225]. Similarly, other vasoactive amines are increased in the plasma of laminitis-prone ponies placed on lush pasture [255]. Notably, another member of *Negativicutes* isolated from the equine cecum, *Allisonella histaminiformans*, is able to produce histamine from histidine [256]. Thus, the excess in readily available energy drives the proliferation of LAB, which produce lactic acid used by *Negativicutes*, which in turn produce histamine and other vasoactive compounds that contribute to inflammation in the hoof.

Changes in the fecal microbiome of horses with naturally occurring chronic laminitis are less clear and characterized by minimal differences in beta-diversity or RA of core taxa [257]. Interestingly, however, the fecal microbiome of horses with chronic laminitis was associated with greater richness compared to healthy controls [257], reminiscent of the aforementioned differences between obese and non-obese control horses [69,245]. Obesity and reduced insulin sensitivity have been identified as risk factors for pasture- and endocrinopathy-associated laminitis in horses [258,259], and this idiosyncratic effect on richness raises the question of common microbiological effects or mechanisms in these conditions.

Osteoarthritis in horses is associated with increased RA of several members of *Bacillota*, *Akkermansia*, and *Campylobacter* and decreased RA of *Clostridium* and *Fibrobacter*, independent of age and BCS [260].

#### 6.2.3. Immune-Mediated Conditions and Hypersensitivities

In horses with asthma and healthy controls removed from pasture and introduced to either good- or poor-quality hay to induce airway obstruction and inflammation, the microbiomes present in the upper and lower airways are influenced by the level of antigen exposure [9]. However, only the lung microbiome differs between asthmatic and healthy horses, and only under inflammatory conditions, suggesting it is a reflection of the asthmatic event rather than triggering factor. In contrast, the fecal microbiome of horses under similar conditions does not differ between asthmatic and healthy horses and changes very little during antigen exposure [158]. A study comparing horses with severe asthma or *Culicoides* hypersensitivity and healthy horses in the same barn found significant effects of environmental factors (e.g., access to pasture) but no difference in diversity or composition of the fecal microbiome based on clinical signs [182]. Equine recurrent uveitis (ERU) is a multifactorial immune-mediated condition triggered by bacterial antigens. As above, no differences were detected in any metric between horses affected with ERU and healthy controls [261].

#### 6.2.4. Plant-Based and Environmental Toxins

Various toxins, when ingested, induce changes in the fecal microbiome. Equine Grass Sickness, due to ingestion of soilborne *Clostridium botulinum* producing type C toxins, induces gut paralysis and severe dysbiosis characterized by increased RA of phyla *Bacteroidota* and reduced RA of phyla *Bacillota* [262]. The microbiome of horses affected with atypical myopathy, induced by intoxication with protoxins in *Acer* (i.e., Maple) and related seeds and seedlings, is also substantially different compared to unaffected co-grazing horses [263,264]. The ability of maple toxins to enter the milk of lactating mares [265,266] suggests the microbiome of suckling foals may also be affected. Other phytotoxins capable of disrupting GIT motility [e.g., white snakeroot (*Ageratina altissima*), nightshades (*Solanum* spp.)] would very likely result in similar changes in the composition of the microbiome.

### 6.3. Iatrogenic and Management-Related

#### 6.3.1. Antimicrobial Drugs

In the context of iatrogenic dysbiosis, antimicrobial drugs are the greatest area of concern. In a retrospective multi-center survey of 26 equine veterinary hospitals on five continents, 55% (792/1419) of horses presenting with acute diarrhea received antimicrobial drugs within the first 24 h of admission, often without a clear indication [267]. Despite its potential effect on *Salmonella* shedding [268], oxytetracycline is the most commonly prescribed antimicrobial for horses with acute diarrhea in North America, whereas penicillin and gentamicin are more commonly used to treat diarrhea in horses elsewhere in the world [267]. Overall, the most commonly prescribed antimicrobials for horses worldwide are TMS, penicillin, gentamicin, oxytetracycline, and doxycycline [269,270,271]. The gut microbiome of horses is a natural reservoir for antimicrobial resistance genes [272], associated with antibiotics produced by soil microbes (e.g., *Streptomyces* spp.) [89,146]. However, domestic horses possess higher levels of ARGs than feral horses, including greater levels of tetracycline resistance genes [146], collectively suggesting that current management practices are associated with increased antibiotic resistance in horses at the population level.

In addition to the aforementioned antimicrobial stewardship and public health considerations, there is a high likelihood of “off-target” bactericidal activity that may or may not affect horse well-being. Antibiotics exert a range of effects on the equine fecal microbiome depending on drug class and route of administration, but reduced richness and alpha-diversity are common outcomes [227,273,274,275,276,277,278,279,280,281,282]. As such, the reduction in richness or Shannon diversity can be used to compare the effect of various antibiotics on the equine fecal microbiome (Figure 4). In studies examining the effect of antimicrobials on the equine microbiome, there are several notable consistencies, despite the variability in study design and technical details. First, parenterally administered antimicrobials tend to have a less substantial impact on richness and alpha-diversity than orally administered drugs. Within the β-lactams, ceftiofur [274,276] has an apparently greater effect on fecal richness than penicillin (with [274] or without gentamicin [279]), potentially due to partial biliary elimination of ceftiofur [283,284]. While not included in Figure 4 due to its study design, recent work suggests that cephalothin sodium (20 mg/kg, IV, TID × 5 days), a first-generation cephalosporin useful against *Staphylococcus* and *Streptococcus*, has no detectable effect on richness or RA of core taxa within the equine microbiome [203]. Among orally administered drugs, TMS appears to have the least impact on richness [273,274,275,278,285,286], while rifampin [287], metronidazole [275,280,281], doxycycline, and erythromycin [275] have increasingly potent effects on richness. These relative effects are reflected in the fecal richness of 32 horses with dysbiosis associated with clinical use of antibiotics. Horses treated with TMS or penicillin and gentamicin were least affected (or unaffected), horses treated with ceftiofur or metronidazole were more affected, and horses treated with doxycycline were the most severely affected in terms of fecal richness [227]. Clioquinol, a potent chelator of Cu^2+^ and Zn^2+^ used to treat suspected protozoal diarrhea and fecal water syndrome, induces a dramatic 90% reduction in fecal richness [282], possibly due to the dependence of several CAZymes on Zn^2+^ or Cu^2+^ [90,92]. Highlighting the value of benign neglect, even the most severely depleted microbiomes are no different from control or baseline samples following discontinuation of the drug and a recovery period [275,282,288]. The effects of antibiotics are selective and may be more severe in cecum than in feces [280]. The phyla *Verrucomicrobiota*, *Kiritimatiellaeota*, and *Fibrobacterota* are particularly sensitive to multiple antibiotics [142,273,280]. Antibiotic-associated diarrhea is associated with increased RA of *Bacteroidota* and *Pseudomonadota* and reduced RA of *Verrucomicrobiota* [142], the latter distinguishing it from antibiotic use without diarrhea [227]. Within *Pseudomonadota*, pathogenic organisms are often enriched. *Enterobacteriaceae* are enriched in the feces of foals after 7 days of treatment with ampicillin and amikacin or gentamicin [289], and *Salmonella enterica* can be isolated from the cecum of horses following seven days of metronidazole administration [280]. Similarly, *C. difficile* and *C. perfringens* are readily isolated from the feces of adult horses following treatment with ceftiofur, penicillin and gentamicin, or sulfamethoxazole-trimethoprim [277]. Virtually all antimicrobials used in equine practice are also associated with the rapid development of antimicrobial resistance (AMR), as AMR *E. coli* was isolated from over 60% (138/228) of antibiotic-treated horses [290].

#### 6.3.2. Other Pharmacologic Agents

Other classes of drugs can also alter the equine microbiome, albeit to a lesser degree than most antimicrobials. For example, non-steroidal anti-inflammatory drugs (NSAIDs), the most commonly prescribed class of drugs in horses, induce significant dysbiosis in horses. Phenylbutazone (4.4 mg/kg, PO, SID) and firocoxib (0.1 mg/kg, PO, SID) both induce a roughly 25% reduction in richness by the 10th day of administration [291], accompanied by changes in the fecal metabolome [292] and a transient compromise in gut epithelial barrier function [293].

Treatment with anthelmintics, including moxidectin, moxidectin and praziquantel, fenbendazole, and pyrantel, has been associated with a significant reduction in alpha-diversity [294,295] and modest changes in beta-diversity [8] or RA of core taxa [220,296]. As with NSAIDs, however, this effect on alpha-diversity is transient, as are the changes in RA of several taxa [294]. Other pharmaceuticals commonly used in horses with no detectable effect on the fecal microbiome include omeprazole [297,298], misoprostol [299], and metformin [300].

#### 6.3.3. Physical and Psychological Stress and Behavior

Factors and events presumed to be psychologically stressful for horses can induce detectable changes in the fecal microbiome [301]. Notably, a one-hour transport induces significant change in the hindgut microbiome [302], purportedly greater than that induced by a 12 h fast or 6 h anesthetic procedure including premedication with medetomidine and phenylbutazone, induction with ketamine and diazepam, and maintenance with isoflurane, medetomidine, LRS and hydroxyethyl starch infusions, highlighting the substantial impact of transport on horses [303].

Among 41 different variables in a study of 185 healthy sport horses, the specific equitation discipline explained the greatest amount of variability in the fecal microbiome [304]. Notably, the same study identified significant correlations between the RA of specific genera and several behavioral “indicators of a compromised welfare state” [304], including oral and locomotive stereotypies and aggressiveness. Noteworthy associations include increased RA of *Helicobacter* and *Acinetobacter* with oral stereotypies and increased RA of *Streptococcus* with aggressiveness. The greater RA of *Streptococcus* induced by a high-starch diet is also associated with greater reactivity in horses faced with unfamiliar events, suggesting an influence of the microbiome on behavior [305,306,307]. In a study comparing the fecal microbiome of horses with crib-biting (i.e., cribbing, an oral stereotypy) and sex-, breed-, and age-matched horses in the same herd, cribbing was associated with greater RA of family *Ruminococcaceae*. While not reported by the authors, the data also suggest an association between cribbing and the RA of *Streptococcus* [308]. A study of trigeminal-mediated head-shaking in horses failed to detect meaningful differences between affected horses and unaffected controls [309]. Diet and stress-induced changes in the microbiome can be remedied, at least partially, by periods at pasture with conspecifics [310].

Pregnancy and lactation represent physical stresses to mares, jennies, and mollies. While pregnancy does not appear to be associated with significant changes in alpha- or beta-diversity of the fecal microbiome of mares [207,311], there may be changes in the RA of certain taxa including *Akkermansia*, *Moraxellaceae*, and *Enterobacteriaceae*, some of them occurring at different stages of pregnancy [312,313]. Two studies in Dezhou donkeys suggest that changes in the fecal microbiome of pregnant jennies are most pronounced during the first trimester [312,314].

## 7. Therapies and Preventive Care Targeting the Gut Microbiome

### 7.1. Probiotics

Probiotics are defined as live microbes with health benefits when ingested, prebiotics are the metabolic substrates used by probiotic organisms, and postbiotics are the beneficial metabolites produced by probiotic organisms. A recent survey of 26 equine veterinary hospitals on five continents reported that probiotics were administered to 15% (215/1438) of horses admitted for acute diarrhea [267]. The most common organisms in equine probiotics are LAB (*Lactobacillus*, *Bifidobacterium*, *Enterococcus*, *Pediococcus*) and the yeast *Saccharomyces*. That said, studies suggest that only a small minority of products in the equine market meet their label claims [315,316].

Studies testing the ability of bacterial probiotics to reduce the incidence of diarrhea in newborn foals have yielded mixed results, complicated by the fact that few used the same microbes. While probiotics containing *Bacillus cereus* var. *toyoi* had no detectable effect on diarrhea or pathogen burden in foals [317], probiotics containing *L. pentosus*; *L. rhamnosus* and *E. faecium*; or *L. rhamnosus* (two strains), *L. plantarum* (two strains), and *B. animalis lactis* were all associated with greater frequency or severity of diarrhea in foals [318,319,320]. Assessments of the foal microbiome pre- and post-supplementation suggest a limited ability of probiotics to change the composition of the fecal microbiome in foals [321,322]. In contrast, two studies testing the effects of probiotic cocktails derived from multiple LAB species isolated from healthy horses reported significant reductions in the frequency and duration of diarrhea in foals receiving probiotics, relative to placebo-treated foals [323,324]. The probiotics tested in these latter studies were more diverse, containing *L. equi*, *L. reuteri*, *L. johnsonii*, *L. crispatus*, and *L. salivarius*, or *L. equi*, *L. reuteri*, *L. johnsonii*, *L. ruminis*, and *B. boum*, but the reasons for the discrepant findings described above are unclear.

In healthy adult horses, probiotics provide negligible benefit to the microbiome during recovery from antimicrobial challenge [285,286]. This aligns with other studies finding no effect of probiotics on alpha- or beta-diversity of the fecal microbiome in healthy horses [325,326]. Despite the lack of observable effect on composition, certain probiotics may provide beneficial effects, nonetheless. A strain of *Bacillus coagulans* provides robust and reliable protection from exercise-induced inflammation [327].

There is also limited evidence of benefit from probiotics in terms of pathogen shedding. One study reported a 65% reduction in *Salmonella* shedding in hospitalized horses; however, horses were hospitalized for non-gastrointestinal complaints and showed no clinical signs associated with *Salmonella* infection. As such, the 65% reduction represented the difference between 4 and 11.5 shedding incidents per 1000 horse-days at risk in the probiotic- and placebo-treated horses [328]. Two comparable studies performed in more clinically relevant populations (i.e., horses hospitalized for colic) failed to detect an effect of commercially available probiotics on *Salmonella* shedding, prevalence of diarrhea, or length of hospitalization [329,330].

There is a growing body of data supporting the health benefits of probiotics containing *Saccharomyces*, including *S. cerevisiae* and *S. boulardii*, in horses. Administration of live *S. cerevisiae* to horses receiving a high-fiber or high-starch diet is associated with increased fibrolytic activity, increased RA of multiple *Bacillota*, *Bacteroidota*, and *Fibrobacter*, and reduced RA of *Streptococcus* [166] and *Enterobacteriaceae* [65,331]. While a postbiotic *S. cerevisiae* fermentation product showed no detectable effect on the fecal microbiome of Thoroughbred racehorses [332], other studies suggest similar postbiotics stabilize the hindgut microbiome during stressful events [301].

### 7.2. Prebiotics and Botanicals

Prebiotics are typically non-digestible carbohydrates from plant fiber that serve as the substrate for endogenous beneficial bacteria. Cellobiose as a prebiotic is associated with a dose-dependent increase in the ratio of *Bacillota* to *Bacteroidota* and increased RA of order *Coriobacteriales* and genus *Clostridium* [333]. Supplementation with mannan oligosaccharides (MOS) or the mannan-rich fraction of yeast (*S. cerevisiae*) cell wall are associated with greater richness and alpha-diversity and enrichment of SCFA-producing bacteria from the *Bacillota* and *Bacteroidota* [334,335]. Feeding an oligosaccharide-rich prebiotic (containing MOS and fructooligosaccharides, FOS) to mares and foals from four weeks pre-partum to day 49 of age resulted in a significant increase in the RA of *Akkermansia* [336]. Interestingly, supplementation of donkey foals with yeast polysaccharides was associated with significantly increased RA of *Streptococcus*, *Lactobacillus*, and *Escherichia-Shigella*, suggesting the possible enrichment of LAB or suppression of beneficial bacteria [337]. Similarly, *Lactobacillus* was enriched in healthy adult horses supplemented with Jerusalem artichoke meal, a source of FOS and inulin [152]. Other studies investigating the effects of psyllium fiber [338], aleurone [339,340], and sugar beet pulp [341] failed to detect significant effects on alpha- or beta-diversity and found limited effects on the RA of core members or the fecal microbiome. Similarly, botanical extracts including proprietary blends and bamboo leaf extracts have been associated with subtle changes in the RA of several high-level taxa, but no effect on overall alpha- or beta-diversity [342].

### 7.3. Fecal Microbiota Transfer (FMT)

Described 20 years ago as an adjunct treatment for *C. difficile* infection in horses [343] and now used by a majority of equine practitioners in some countries [344], protocols have now been proposed for the preparation and administration of FMT in horses [345,346]. While the effects of short-term freezing of FMT material are minimal [347,348], use of fresh feces is recommended as long-term storage and cryoprotectants may affect the viability or metabolic activity of microbes [348,349,350]. Following a brief pre-treatment with oral antacids (e.g., omeprazole), a fecal slurry prepared from a healthy donor horse is administered to the recipient via nasogastric tube.

The efficacy of FMT to treat spontaneously occurring enteric conditions is still in question, as many prior studies failed to include healthy controls [351,352,353] or detect any clinical benefit of FMT [235,354,355] in the treatment of colitis [353], diarrhea [351,352,354,355], and FFWS [235]. Data supporting the efficacy of FMT come from a study of 12 adult horses with colitis that reported modest benefits of FMT in reduction of diarrhea and day-to-day improvement [180]. Moreover, earlier work lacking a control group reported that, following treatment of diarrhea via FMT, clinical response was associated with increased alpha-diversity and RA of *Verrucomicrobiota* [351].

Studies have also investigated the use of FMT to prevent or restore dysbiotic changes following pharmacological insult or dietary challenge. Perhaps not surprisingly, FMT is unable to prevent dysbiosis induced by concomitant administration of metronidazole [281]. Similarly, FMT from a healthy donor yielded little benefit relative to vehicle-treated controls following TMS-induced dysbiosis [356]. While the findings from the latter study may reflect the small sample size (*n* = 3/group), they may also reflect the natural ability of the hindgut microbiome to normalize and re-equilibrate following challenge or insult. Alternatively, FMT significantly accelerates the return to health following oligofructose-induced diarrhea, in terms of microbiome composition, clinical parameters (body temperature, stool consistency), and inflammatory markers [226].

## 8. Conclusions

Horses and their gut microbiome have co-evolved, and it is not possible to fully appreciate equine physiology absent the gut microbiome. While many intrinsic factors and disease processes can subtly shape the microbiome or cause dysbiosis in horses, serious health-threatening concerns frequently arise from human interventions. Considerations regarding major risk factors for dysbiosis and best practices for maintenance of a healthy gut microbiome follow.

### 8.1. Antimicrobial Stewardship

The need for vigilant antimicrobial stewardship in horses cannot be overemphasized. From the standpoint of host health, antimicrobials are likely to inhibit growth or kill more than just the target pathogen. The microbiome has redundancies, but sufficient loss of core microbes will eventually lead to reduced functionality and increased susceptibility to other pathogens. In terms of global health, the fecal metagenome of horses and livestock constitute rich reservoirs of ARGs. While many ARGs are expressed intrinsically, even in the microbiome of wild or feral animals, many others are induced by exposure to antimicrobials and transferred horizontally among gut commensals via plasmid-mediated conjugation, bacteriophages, or other methods [357]. The presence of ARGs in fecal microbes also provides those bacteria a competitive advantage in the soil [287], making this a One Health issue of concern to animals, humans, and the environment.

### 8.2. Forage, Freedom, and Friends (The Three Fs) and the Microbiome

In horses, the gut microbiome is inherently related to animal welfare, in that the maintenance of a healthy gut microbiome is dependent on the same three things responsible for their physiological and psychological welfare—forage, freedom, and friends [358]. Horses consume roughly 2% of their body weight in forage daily. Among grasses, legumes, and pelleted chows, the dietary preference of horses always correlates with greater carbohydrate (particularly NSC) or protein content [359,360,361,362], suggesting forage is selected based on both nutrient content and palatability. Achieving an appropriate level of nutrition on pasture alone thus depends on the presence of adequate vegetation containing good nutrient content and the freedom to search for the forages delivering the highest carbohydrate and protein content. Free-ranging horses travel several kilometers and spend over 16 h per day in their quest for forage [363]. Freedom of movement also promotes physical activity and avoids the psychological stress of confinement. Lastly, horses are highly social animals, and the presence of multiple healthy horses ensures continuous re-inoculation of the environment with gut microbes which are then acquired via inadvertent acquisition of bacterial cells or spores while grazing or even overt coprophagy. The relevance of pasture-mates to the microbiome is clear, as social interactions between horses lead to increased similarity in microbiome composition [364]. The combined value of forage, freedom, and friends is perhaps best demonstrated by studies wherein severely depleted fecal microbiomes are completely restored in terms of richness and beta-diversity following return to pasture with other horses and benign neglect for several weeks [282].

In the real world, however, there are often limitations to what is available, or restrictions on one or more of the three Fs are necessary due to the use of the horse. When access to fresh forage is limited, supplementation with dried forage or concentrates is needed to achieve an appropriate level of nutrition. While the healthy equine microbiome is resilient when faced with minor or gradual changes in dietary content, there is a point at which replacement of forage with NFC and simple sugars may contribute to hindgut acidosis, proliferation of *Streptococcus*, and adverse health outcomes. The growing number of available concentrates and supplements is a double-edged sword, and horse owners and veterinarians alike are encouraged to work with an animal or veterinary nutritionist when questions arise regarding the sufficiency of an equine diet plan.

Additional research is needed to identify and characterize other iatrogenic and management-related causes of dysbiosis, to develop and refine effective methods of mitigating dysbiosis when it occurs, and to develop microbiome-based tools that provide clinically actionable data to veterinarians and horse owners. Many horses receive nutritional supplements beyond their daily requirements. Just like carbohydrates, protein, and trace minerals, every nutrient essential for the microbiome has an upper limit, at which point it begins to negatively impact microbial function and/or host health. Thus, there is also a need for additional *in vivo* research into the effects of the wide range of nutritional supplements given to horses on the microbiome and relevant health outcomes. Ultimately, the health of a horse is reliant on the health of its microbiome, and additional research is needed to generate well-informed guidelines for horse owners and caretakers.

## Figures and Tables

**Table 1:** Table showing the approximate reported Age of Microbiome Maturation (AMM) in weeks, Mean Estimated Lifespan (MEL) in years, and Lifespan-adjusted Time to Maturation (LTM) expressed as a percentage, in eight different terrestrial mammals listed in order of increasing lifespan. References for AMM times are provided in the column at right.

Animal	AMM (Weeks)	MEL (Years)	LTM (%)	References
*Mus musculus*	2.5	1	4.81%	[39]
*Oryctolagus cuniculus*	9	8	2.16%	[40,41]
*Canis lupus familiaris*	26	11.5	4.35%	[42,43]
*Felis catus*	36	15	4.62%	[44]
*Bos taurus*	26–52	15	3.33–6.66%	[45,46]
*Sus scrofa*	20	15	2.56%	[47,48]
*Equus caballus*	8–13	30	0.54–0.83%	[27,29,31,32,33,34,35]
*Homo sapiens*	130–156	73	3.42–4.11%	[49,50]

## Data Availability

No new data were created or analyzed in this study.

## Supplementary Materials

The following supporting information can be downloaded at: https://www.mdpi.com/article/10.3390/vetsci13070659/s1, Table S1: Phyla, families, and genera reported as part of the core microbiome in multiple studies, grouped alphabetically. Colored taxa are commonly reported as diet-sensitive markers, with taxa shaded in green often enriched by intake of forage (pasture and hay) and taxa shaded in pink often enriched during intake of cereal grains and concentrates.

## Abbreviations

The following abbreviations are used in this manuscript:

ARG	Antimicrobial resistance gene
BA	Bile acids
BCS	Body condition score
CP	Crude protein
EGUS	Equine gastric ulcer disease
FEC	Fecal egg count
FFWS	Free fecal water syndrome
FMT	Fecal microbiome transfer
FOS	Fructooligosaccharides
GIT	Gastrointestinal tract
IBD	Inflammatory bowel disease
LAB	Lactic acid bacteria
MOS	Mannan oligosaccharides
NDF	Neutral detergent fiber
NFC	Non-fibrous carbohydrate
RA	Relative abundance
SCFA	Short-chain fatty acids
VFA	Volatile fatty acids
