# Supplementary material for: A Comprehensive Review of the Equine Gut Microbiome in Health and Disease
Source: Vet Sci. 2026 Jul 7;13(7):659. doi: 10.3390/vetsci13070659 (PMC13431609; doi:10.3390/vetsci13070659)
Supplement: Supplementary file 1 [file vetsci-13-00659-s001.zip › vetsci-4376936-supplementary.pdf]

Table S1. Phyla, families, and genera reported as part of the core microbiome in multiple studies, grouped alphabetically. Colored taxa are commonly reported as diet-sensitive markers, with taxa shaded in green often enriched by intake of forage (pasture and hay), and taxa shaded in pink often enriched during intake of cereal grains and concentrates.

Phylum	Family	Genus	References
**Actinomycetota (HGCGP)**	*Coriobacteriaceae*		[54,67,70,73]
	Eggerthellaceae	multiple	[69,70,72,73]
Bacillota **(LGCGP)**	*Acidaminococcaceae*	*Acidaminococcus*	[38,68,72]
		Phascolarctobacterium	[6,7,54,68,69,71,74]
	Anaerovoracaceae	*Anaerovorax*	[69,70]
		Mogibacterium	[6,54,69,72]
		multiple others	[7,30,38]
	Christensenellaceae	multiple	[6,7,38,54,69,70]
	UC *Clostridiales*		[30,54]
	Clostridiaceae	*Anaerosporobacter*	[68,69,72]
		Clostridium	[7,54,68,69,73,74]
		multiple others	[7,38,54,68]
	Erysipelotrichaceae	UCG-004	[6,71]
		multiple others	[38,54,67-69]
	Eubacteriaceae	*Eubacterium*	[6,7,38,54,68,72-74]
	Hungateiclostridiaceae		[7,38,73]
	Lachnospiraceae (Clostridium cluster XIVa)	*Blautia*	[6,7,54,68,70,72]
		Butyrivibrio	[7,73,74]
		Coprococcus	[7,54,68,70,72,73]
		Dorea	[54,68]
		Intestinimonas	[7,69]
		Lachnoclostridium	[7,69]
		Pseudobutyrivibrio	[6,7,54,68,70,72,73]
		Roseburia	[7,54,68,72]
		multiple others	[6,7,54,68,69]
	Lactobacillaceae	*Lactobacillus*	[54,68,72-74]
	Leuconostocaceae		[54,70]
	Oscillospiraceae	*Oscillibacter*	[7,68-70,72]
		Pseudoflavonifractor	[7,69]
		multiple others	[7,38]
	Ruminococcaceae (Clostridium cluster IV)	*Faecalibacterium*	[7,68,72,73]
		Oscillospira	[30,54,70,73]
		Papillibacter	[7,68,72,74]
		Ruminococcus	[6,7,54,68-70,72-74]
		Saccharofermentans	[6,7,70]
		Sporobacter	[7,68,69,72-74]
		UCG-002	[6,71]
		UCG-010	[6,71]
		multiple others	[6,7,68]
	Streptococcaceae	*Streptococcus*	[54,70-74]

	Selenomonadaceae	*Schwartzia*	[7,72]
		multiple others	[54,73,74]
	Veillonellaceae		[54,74]
Bacteroidota	UC *Bacteroidales*	BS11 group	[6,30,54]
		multiple others	[38,67,69]
	Bacteroidaceae	BF311	[30,54,70]
		Bacteroides	[54,73,74]
	Paludibacteraceae	*Paludibacter*	[7,30,54,68,70]
		RF16 group	[6,30,38,54]
	Paraprevotellaceae	*Paraprevotella*	[7,54,68-70]
		YRC22 group	[30,54,70]
	Porphyromonadaceae	*Porphyromonas*	[6,30,70,73]
	Prevotellaceae	*Alloprevotella*	[69-71,73]
		Prevotella	[6,54,68-70,72-74]
		UCG-001	[6,38,71]
		UCG-004	[6,38]
		multiple others	[6,67]
	Rikenellaceae	RC9 group	[6,38,54,70,71]
	Tannerellaceae	*Parabacteroides*	[7,73]
Cyanobacteria	*Vampirovibrionaceae*	*Vampirovibrio*	[7,69]
Fibrobacterota	*Fibrobacteraceae*	*Fibrobacter*	[6,38,54,67-70,72,74]
Kiritimatiellaeota	WCHB1-41 group		[6,30,38,68,72]
	RFP12 group		[30,54]
Mycoplasmatota	*Anaeroplasmataceae*	*Anaeroplasma*	[54,68,69,74]
		Asteroleplasma	[54,69]
	Mycoplasmataceae	*Mycoplasma*	[54,71,73]
		multiple others	[30,38,54,69]
Pseudomonadota	UC *Alphaproteobacteria*		[30,54]
	Pasteurellaceae	*Actinobacillus*	[54,72]
Spirochaetota	*Spirochaetaceae*	*Treponema*	[6,7,30,38,54,68-74]
Verrucomicrobiota	*Verrucomicrobiaceae*	*Akkermansia*	[6,54,68,71,73,74]
Euryarchaeota	*Methanobacteriaceae*	*Methanobrevibacter*	[6,54,74]
Methanobacteriota	*Methanocorpusculaceae*	*Methanocorpusculum*	[6,54,68,74]
